# Compliance with mass marketing solicitation: The role of verbatim and gist processing

**DOI:** 10.1002/brb3.2391

**Published:** 2021-10-17

**Authors:** Julia Nolte, Yaniv Hanoch, Stacey A. Wood, Valerie F. Reyna

**Affiliations:** ^1^ Department of Human Development Cornell University Ithaca New York USA; ^2^ Department of Psychology Scripps College Claremont California USA; ^3^ Department of Psychology Cornell University Ithaca New York USA; ^4^ Southampton Business School University of Southampton Southampton UK

**Keywords:** consumer fraud, fuzzy‐trace, mass marketing solicitation

## Abstract

**Introduction:**

Mass marketing scams threaten financial and personal well‐being. Grounded in fuzzy‐trace theory, we examined whether verbatim and gist‐based risk processing predicts susceptibility to scams and whether such processing can be altered.

**Methods:**

Seven hundred and one participants read a solicitation letter online and indicated willingness to call an “activation number” to claim an alleged $500,000 sweepstakes prize. Participants focused on the solicitation's verbatim details (hypothesized to increase risk‐taking) or its broad gist (hypothesized to decrease risk‐taking).

**Results:**

As expected, measures of verbatim‐based processing positively predicted contact intentions, whereas measures of gist‐based processing negatively predicted contact intentions. Contrary to hypotheses, experimental conditions did not influence intentions (43% across conditions). Contact intentions were associated with perceptions of low risk, high benefit, and the offer's apparent genuineness, as well as self‐reported decision regret, subjective vulnerability to scams, and prior experience falling for scams.

**Conclusions:**

Overall, message perceptions and prior susceptibility, rather than experimental manipulations, mattered in predicting scam susceptibility.

## Abstract 2

Seven hundred and one participants read a solicitation letter online and indicated willingness to call an “activation number” to claim an alleged $500,000 sweepstakes prize. Participants focused on the solicitation's verbatim details (hypothesized to increase risk‐taking) or its broad gist (hypothesized to decrease risk‐taking). Experimental conditions did not influence contact intentions (43% across conditions); instead, intentions were associated with perceptions of low risk, high benefit, and the offer's apparent genuineness, as well as self‐reported decision regret, subjective vulnerability to scams, and prior experience falling for scams.

## 1 INTRODUCTION

Mass marketing scams (MMS) carried out online, via snail mail, or other mass‐communication methods are becoming increasingly common: In the United States alone, more than 1.7 million fraud complaints were registered in 2019, culminating in a loss of $765 million (Federal Trade Commission, 2020). Beyond their financial ramifications, MMS can also result in long‐term physical and/or emotional suffering for the victims (Button, Lewis, et al., 2014; Shichor et al., 1996). As a result, MMS pose a substantial threat to consumers’ financial and personal well‐being.

Because response rates to MMS can be as high as 58% (Modic & Lea, 2013; Wood et al., 2018), many government organizations recommend steps that the public can take to protect themselves against MMS. These steps include ignoring emails or letters sent by strangers, not divulging personal information over the phone, and not claiming rewards won through prize drawings people do not remember entering. Despite these warnings, scammers are remarkably successful in using these unsolicited communications to identify individuals who are responsive to MMS, and then targeting them more directly, with the aim of extracting money (Button, McNaughton Nicholls, et al., 2014; Titus & Gover, 2001). For example, individuals may be lured into paying an advance fee for a supposed prize, sham charities, or bank account schemes (e.g., Titus et al., 1995).

### 1.1 Fuzzy‐trace theory

Decision makers’ willingness to comply with financial fraud may be influenced by the way they process risk‐related information. According to fuzzy‐trace theory, information is encoded in multiple representations that vary in precision (Reyna, 2008). Gist representations reflect the bottom‐line meaning of information, for instance whether the scam advance fee (say, $50) is perceived as “low” or “high”. Verbatim representations encode objective, surface‐level information such as precise wording and numbers.

Gist‐based reasoning typically operates on the simplest representation that accomplishes a decision, such as “gaining something” versus “possibly gaining something or nothing” (categorical some‐none gist). Making more precise risk‐reward tradeoffs (by using more exact gist‐based or verbatim‐based comparisons) promotes risk seeking when benefits appear high and risks negligible. Such risk takers are more likely to endorse the more precise ordinal gist of “less risk is better than more risk” but not the categorical gist that “no risk is better than some risk” for the same risky behaviors, and risk avoiders do the opposite, despite the fact that both endorsements express risk aversion (Mills et al., 2008). Consequently, categorical risk perception negatively predicts risky intentions and behavior, in contrast to more precise measures of risk perception (e.g., quantitative estimates) that positively predict (i.e., reflect) risky intentions and behavior.

In the context of online risk‐taking, White and colleagues (2017) evaluated how measures of verbatim and gist‐based reasoning relate to people's engagement in risky behaviors that increase susceptibility to internet fraud (e.g., sharing personal information and chatting with strangers online). Results confirmed that verbatim reasoning was tied to embracing online risks, whereas gist‐based reasoning was associated with risk avoidance. This echoes earlier findings that have also linked the disclosure of sensitive information online to verbatim rather than gist‐based reasoning (e.g., White et al., 2015).

### 1.2 The current study

To test whether susceptibility to fraud can be experimentally altered, participants were confronted with a foot‐in‐the‐door scam meant to identify individuals who are generally vulnerable to MMS (i.e., call an “activation number” to claim an alleged sweepstakes prize). Each participant was randomly assigned to an instructional condition that either aimed at inducing verbatim or gist‐based processing. Extrapolating from fuzzy‐trace theory, we hypothesized that participants in the verbatim condition would be more interested in calling the “activation number” than participants in the gist condition.

Earlier work suggests that risk and benefit perceptions are central to individuals’ engagement in risky behaviors such as responding to scams (Hanoch et al., 2006; Wood et al., 2018). Therefore, we hypothesized that higher benefit and lower risk perceptions would predict stronger intentions to respond to the solicitation. Because risk and benefit perceptions are often inversely related to each other (e.g., Finucane et al., 2000) and we assumed that focusing on the verbatim details of the solicitation (e.g., the dollar amount of the prize participants had “won”) would increase benefit perceptions, we hypothesized that risk perceptions would be lower and benefit perceptions higher in the verbatim condition.

Furthermore, we assessed a range of individual difference measures past research has identified as important correlates of risk‐taking preferences or susceptibility to fraud, including trait‐based decision regret concerning forgoing opportunities (Nordgren et al., 2007; Richard et al., 1996) and how strongly participants consider possible consequences of their actions (Murphy & Dockray, 2018). Financial literacy (James et al., 2014) and financial risk tolerance (Anderson, 2013; Van Wyk, 2001) were included as known correlates of fraud susceptibility. Participants were also asked to provide a subjective assessment of their own susceptibility to scams (see Langenderfer & Shimp, 2001; Mueller et al., 2020) and to self‐reported past experiences with financial fraud (Schoepfer & Piquero, 2009; Titus & Gover, 2001). Finally, we included measures of verbatim and gist‐based risk perception concerning letter or phone scams, with the expectation that higher scores on verbatim [gist] measures would positively [negatively] predict willingness to respond to the presented solicitation (White et al., 2015).

## 2 METHOD

The study design was approved by the Faculty Research Ethics Committee at Plymouth University. Upon reasonable request addressed to Julia Nolte ( jn472@cornell.edu ), data can be made available to other authors.

### 2.1 Participants

We recruited *N* = 840 US citizens through Amazon's Mechanical Turk (MTurk), which generates reliable data similar to data collected in more conventional settings (e.g., Mortensen & Hughes, 2018;). *N* = 139 participants were excluded due to missing data or failed attention checks. This resulted in a final sample of *N* = 701 participants (18–78, *M*_age_ = 38.15, SD_age_ = 11.96).

### 2.2 Material

Participants perused a solicitation letter (adapted from Wood et al., 2018, high authority/high scarcity version with no advance fee) informing them that they had been entered into prize drawing after shopping with one of several well‐known companies. In order to claim their $500,000 prize, participants were urged to call an “activation number” before their claim expired.

Participants were randomly assigned to one of two counter‐balanced conditions (see Appendix A). The verbatim condition instructed participants to focus on the letter's exact details. This condition was designed to induce verbatim reasoning, such as drawing attention to the specificities of the offer (e.g., deadline, contest organizer), including its benefits (i.e., the precise amount of money participants stood to gain), which should promote risk taking. The gist condition instructed participants to imagine that they were to tell friends or family about the letter in one or two sentences, using their own words. This condition made use of strategies central to diverse interventions that have been successful at fostering gist reasoning. Specifically, the gist condition combined elements of strategic attention/inhibition and integrated reasoning/organization (Anand et al., 2011; Cook et al., 2014). Strategic attention and inhibition refer to the differentiation between important and unimportant information, whereas integrated reasoning and organization involve the ability to paraphrase and abstract information using one's own words.

### 2.3 Measures

#### 2.3.1 Demographics

We collected data on participants’ age, race/ethnicity, gender, employment status, income, education, and marital status. Participants also identified their political worldview using a 7‐point Likert scale ranging from (1) Very Liberal to (7) Very Conservative.

#### 2.3.2 Individual difference measures

Decision regret: Trait‐based generalized decision regret was measured using five items such as “when I think about how I'm doing in life, I often assess opportunities I have passed up” (*α* = .80; Schwartz et al., 2002, p. 1882) using a 7‐point Likert scale anchored at (1) Completely disagree and (7) Completely agree.

Outcome focus: Participants’ tendency to consider possible positive (*α* = .89), negative (*α* = .92), or general outcomes (*α* = .91) when making decisions was assessed through the 13‐item elaboration of potential outcomes scale, with items such as “before I make a decision, I consider all possible outcomes” (Nenkov et al., 2007, p. 129) using a 7‐point Likert scale from (1) Strongly disagree to (7) Strongly agree.

Consideration of future outcomes: To what extent participants trade off immediate versus future outcomes was assessed using 12 items including “my convenience is a big factor in the decisions I make or the actions I take” (*α* = .87; Strathman et al., 1994, p. 752) using a 5‐point Likert scale ranging from (1) Extremely uncharacteristic to (5) Extremely characteristic.

Susceptibility to scams: How easily participants fall for scam attempts was measured using a 5‐item self‐report scale, including “if a telemarketer calls me, I usually listen to what they have to say” (*α* = .73; James et al., 2014, p. 4). Response options were anchored at (1) Strongly disagree to (7) Strongly agree on a 7‐point Likert scale.

Financial knowledge: We presented participants with four financial knowledge questions such as “if interest rates rise, what will typically happen to bond prices?” (0–4 points, *α* = .62; Lusardi, 2008).

Financial risk tolerance: Participants’ openness to taking financial risks was measured via five questions such as “I am willing to risk financial losses” (*α* = .89; Jacobs‐Lawson & Hershey, 2005, p. 341) using a 7‐point Likert scale anchored at (1) Strongly disagree and (7) Strongly agree.

History of financial fraud: Participants reported whether they had ever been a victim of financial fraud, whether they had ever received offers similar to the solicitation presented in the present study, and whether they had responded to such an offer.

#### 2.3.3 Fuzzy‐trace theory measures

Following Mills and Reyna (2008), we evaluated individual‐level scam‐related risk perception using three gist‐based measures (categorical risks, global risks, and gist principles) and two verbatim‐based measures (specific and quantitative risks; see Appendix A for these questionnaires).

Categorical (gist) risk perception: To assess participants’ categorical risk perception, we adapted nine “online risk taking” items (e.g., “even low risks, such as identify theft, happen to some people”) to the context of letter or phone scam risks (*α* = .85; White et al., 2015). Participants indicated their agreement with statements on a 5‐point Likert scale ranging from (0) Strongly disagree to (4) Strongly agree.

Global (gist) risk perception: Participants rated the overall risk of (1) giving out personal information or (2) claiming prizes over the phone on a 4‐point scale ranging from (0) None to (3) High (*α* = .82; adapted from White et al., 2015).

Gist principles: Participants indicated which of 13 gist principles concerning the risks of letter/phone scams they endorsed (e.g., “better safe than sorry”), resulting in a sum score between 0 and 13 (*α* = .49; adapted from White et al., 2015).

Specific risk perception: On a 5‐point Likert scale anchored at (0) Very unlikely and (4) Very likely, participants self‐reported their risk of (1) having their personal details stolen and (2) being taken advantage of over the phone within the next two months (*α* = .88; adapted from White et al., 2015).

Quantitative risk perception: Participants rated their likelihood of (1) having had their personal information stolen or (2) having been a victim of a scam on an analogue scale ranging from 0% to 100% (*α* = .89).

### 2.4 Scam‐related measures

Willingness to contact scammers: After reviewing the solicitation, participants rated their likelihood of contact the activation number (adapted from Wood et al., 2018) on a 7‐point Likert scale ranging from (1) Very unlikely to (7) Very likely.

Perceived risks and benefits: Perceived risks and benefits were assessed using 7‐point Likert scales ranging from (1) Very low risks [benefits] to (7) Very high risks [benefits] (Wood et al., 2018).

Perceived genuineness: Participants were asked to indicate whether the solicitation was genuine or a scam (coded as 0 = letter is not genuine, 1 = letter is genuine; adapted from Wood et al., 2018). We also assessed participants’ confidence in their response but omitted confidence from analyses.

### 2.5 Procedure

After providing informed consent, participants perused a solicitation letter and rated its perceived risks and benefits as well as their intentions to respond to the letter. Participants then completed measures of their decision regret, outcome focus, consideration of future outcomes, susceptibility to scams, financial knowledge, financial risk tolerance, history of financial fraud, their perception of the solicitation's genuineness, and demographic background. Finally, participants indicated their agreement with fuzzy‐trace theory measures. Then, participants were debriefed and provided with a link to an external website explaining the risks of MMS.

### 2.6 Analyses

Analyses were conducted in RStudio versions 1.1.423 and 1.3.1093. Because most variables were non‐normally distributed, between‐group comparisons were based on non‐parametric ANOVAs and *X^2^* tests. With almost no exception, comparable results were obtained when conducting Wilcoxon Rank‐Sum tests or parametric tests. Regression results were based on generalized linear models and Nagelkerke's *Pseudo‐R^2^*.

## 3 RESULTS

Three hundred and fifty‐three (50%) participants were randomized to the verbatim condition, 348 (50%) to the gist condition. Descriptive statistics and between‐group comparisons concerning demographic, individual difference, and fuzzy‐trace theory measures are summarized in Table 1. Groups did not differ with regard to most demographic variables but participants in the verbatim condition reported a marginally higher income. With regard to individual difference measures, participants in the verbatim condition reported a significantly stronger positive outcome focus than participants in the gist condition, and a marginally higher general outcome focus.

**TABLE 1:** Descriptive statistics and group comparisons for demographic, individual difference, and fuzzy‐trace theory measures

		Experimental Condition		
	Whole sample	Verbatim	Gist	
	M (SD)/ *n* (%)	M (SD)/ n (%)	M (SD)/ *n* (%)	Group comparison
Demographic measures				
Age	38.15 (11.96)	38.49 (12.20)	37.81 (11.73)	*F*(1, 699) = .46, *p* = .498, *η_p_^2^* = .00
Gender: female	365 (52%)	187 (53%)	178 (51%)	*X^2^* (1, *N* = 696) = .20, *p* = .654
Race/ethnicity: non‐White	187 (27%)	96 (27%)	91 (26%)	*X^2^* (1, *N* = 701) = .05, *p* = .820
Employment status: not full‐time	231 (33%)	106 (30%)	125 (36%)	*X^2^* (1, *N* = 701) = 2.49, *p* = .114
Education^†^	3.42 (1.15)	3.43 (1.20)	3.41 (1.09)	*F*(1, 699) = .01, *p* = .921, *η_p_^2^* = .00
Income^‡^	2.42 (1.09)	2.48 (1.06)	2.35 (1.12)	*F*(1, 699) = 2.98, *p* = .085, *η_p_^2^* = .00
Marital status: not married	360 (51%)	174 (49%)	186 (53%)	*X^2^* (1, *N* = 701) = 1.05, *p* = .305
Political worldview	3.69 (1.90)	3.68 (1.84)	3.70 (1.95)	*F*(1, 699) = .18, *p* = .669, *η_p_^2^* = .00
Individual difference measures				
Decision regret	4.13 (1.33)	4.07 (1.34)	4.20 (1.32)	*F*(1, 699) = 1.51, *p* = .220, *η_p_^2^* = .00
Positive outcome focus	5.18 (1.33)	5.32 (1.24)	5.04 (1.40)	*F*(1, 699) = 5.38, *p* = .021*, *η_p_^2^* = .01
Negative outcome focus	4.20 (1.57)	4.12 (1.57)	4.28 (1.57)	*F*(1, 699) = .43, *p* = .513, *η_p_^2^* = .00
General outcome focus	5.69 (.85)	5.73 (.83)	5.65 (.87)	*F*(1, 699) = 3.75, *p* = .053, *η_p_^2^* = .01
Consideration of future outcomes	3.44 (.70)	3.45 (.69)	3.42 (.71)	*F*(1, 699) = .12, *p* = .727, *η_p_^2^* = .00
Susceptibility to scams	2.36 (1.15)	2.38 (1.15)	2.34 (1.14)	*F*(1, 699) = .71, *p* = .399, *η_p_^2^* = .00
Financial knowledge	2.33 (1.25)	2.37 (1.27)	2.28 (1.23)	*F*(1, 699) = 1.18, *p* = .278, *η_p_^2^* = .00
Financial risk tolerance	3.67 (1.49)	3.70 (1.50)	3.65 (1.48)	*F*(1, 699) = .23, *p* = .631, *η_p_^2^* = .00
History of financial fraud: yes	124 (18%)	61 (17%)	63 (18%)	*X^2^* (1, *N* = 699) = .03, *p* = .852
Has received scam IRL: yes	369 (53%)	181 (51%)	188 (54%)	*X^2^* (1, *N* = 699) = .43, *p* = .514
Has responded to scam IRL: yes^§^	50 (14%)	22 (12%)	28 (15%)	*X^2^* (1, *N* = 369) = .38, *p* = .538
Fuzzy‐trace theory measures				
Categorical risk	3.17 (.54)	3.20 (.52)	3.14 (.56)	*F*(1, 699) = 1.88, *p* = .171, *η_p_^2^* = .00
Global risk	2.41 (.74)	2.39 (.78)	2.42 (.70)	*F*(1, 699) = .06, *p* = .809, *η_p_^2^* = .00
Gist principles	7.97 (1.59)	7.99 (1.68)	7.94 (1.49)	*F*(1, 699) = .19, *p* = .668, *η_p_^2^* = .00
Specific risk	1.18 (1.11)	1.19 (1.09)	1.16 (1.14)	*F*(1, 699) = .53 *p* = .465, *η_p_^2^* = .00
Quantitative risk	30.24 (30.86)	29.04 (30.03)	31.48 (31.68)	*F*(1, 699) = 1.14, *p* = .286, *η_p_^2^* = .00

Abbreviation: IRL, in real life.

^†^Education was coded as 1 = do not have high school degree or GED, 2 = high school degree/GED, 3 = associate's degree, 4 = bachelor's degree, 5 = master's degree, 6 = professional degree (MD, JD, etc.), 7 = PhD.

^‡^Income was coded as 1 = $0–$24,999; 2 = $25,000–$49,999; 3 = $50,000–$74,999; 4 = $75,000–$124,*999*; 5 = $125,000–$174,999; 6 = $175,000+.

^§^ *N* for this variable is 369.

^*^ *p* < .05.

Groups received similar scores on all fuzzy‐trace theory measures. This finding was expected, as the gist‐based fuzzy‐trace theory measures assessed ingrained principles such as “better safe than sorry” or “once someone has your personal details, there is no second chance” unlikely to be changed by our experimental instructions that focused on the message. Similarly, verbatim‐based measures assessed participants’ real‐life likelihood of being or having been the victim of fraud, and were thus not likely to be influenced by our instructions. Due to the lack of group differences with regard to these measures, we did not examine interaction effects of experimental condition and gist‐ and verbatim‐based measures of risk perception on participants’ intentions to contact the scammers.

### 3.1 Group differences in scam‐related measures

Descriptive statistics and between‐group comparisons concerning scam‐related measures are summarized in Table 2. Contrary to expectations, groups did not differ in their willingness to call the action number or perception of how beneficial the offer was. However, participants in the gist condition perceived the offer as marginally riskier than did participants in the verbatim condition. In fact, when conducting parametric tests, we found that participants in the gist condition perceived the solicitation as significantly more risky than participants in the verbatim condition did, *F*(1699) = 4.38, *p* = .037, *n_p_^2^* = .01. In addition, participants in the verbatim condition were marginally more likely to mistake the letter as genuine.

**TABLE 2:** Descriptive statistics and group comparisons for scam‐related measures

		Experimental condition		
	Whole sample	Verbatim	Gist	
	*M* (SD)/ *n* (%)	*M* (SD)/ *n* (%)	*M* (SD)/ *n* (%)	Group comparison
Willingness to call scammers	3.65 (2.24)	3.78 (2.23)	3.51 (2.24)	*F*(1, 699) = 2.54, *p* = .111, *η_p_^2^* = .00
Covariate: income				*F*(1, 699) = 2.67, *p* = .103, *η_p_^2^* = .00
Covariate: positive outcome focus				*F*(1, 699) = 2.12, *p* = .146, *η_p_^2^* = .00
Covariate: general outcome focus				*F*(1, 699) = 2.51, *p* = .114, *η_p_^2^* = .00
Perceived risks	5.14 (1.75)	5.01 (1.83)	5.28 (1.65)	*F*(1, 699) = 3.11, *p* = .078, *η_p_^2^* = .00
Covariate: income				*F*(1, 699) = 3.25, *p* = .072, *η_p_^2^* = .01
Covariate: positive outcome focus				*F*(1, 699) = 3.31, *p* = .069, *η_p_^2^* = .01
Covariate: general outcome focus				*F*(1, 699) = 3.54, *p* = .061, *η_p_^2^* = .01
Perceived benefits	4.16 (2.27)	4.25 (2.26)	4.05 (2.27)	*F*(1, 699) = 1.43, *p* = .232, *η_p_^2^* = .00
Covariate: income				*F*(1, 699) = 1.70, *p* = .192, *η_p_^2^* = .00
Covariate: positive outcome focus				*F*(1, 699) = .84, *p* = .361, *η_p_^2^* = .00
Covariate: general outcome focus				*F*(1, 699) = 1.26, *p* = .261, *η_p_^2^* = .00
Letter is genuine: yes	*n* = 178 (25%)	*n* = 101 (29%)	*n* = 77 (22%)	*X^2^* (1, *N* = 701) = 3.56, *p* = .059

Because participants in the verbatim and gist conditions reported differences in income, general outcome focus, and positive outcome focus, analyses concerning contact intentions, risks, and benefits were repeated as ANCOVAs accounting for the three covariates (Table 2). Results did not meaningfully change.

### 3.2 Predicting willingness to contact scammers

Summarizing across experimental conditions, we regressed participants’ contact intentions on all demographic, individual difference, fuzzy‐trace theory, and scam‐related measures. (Given that 75% of participants realized the letter represented a scam but 43% were willing to respond, complementary analyses predicting genuineness perceptions are reported in Table S1, Appendix B).

#### 3.2.1 Independent entry

In a first step, each predictor was examined separately (Table 3, left columns).

**TABLE 3:** Regression results predicting intentions to respond for each variable entered separately versus all variables entered jointly

	Separate entry		Joint entry			Joint Entry (removing high VIF)
Variables	Β	*Pseudo‐R^2^*	*β*	VIF	Tol.	*β*
Demographic variables						
Age	−.06	.01	.07	1.78	.56	.08
Gender: female	.00	.03	−.03	1.22	.82	−.04
Race/ethnicity: non‐White	.10**	.01	−.03	1.43	.70	−.02
Employment status: not full‐time	−.01	.00	−.01	1.31	.76	.00
Education^†^	.03	.00	−.04	1.35	.74	−.02
Income^‡^	−.02	.00	−.06	1.53	.65	−.04
Marital status: not married	−.07	.00	−.04	1.37	.73	−.04
Political worldview	.09*	.05	−.03	1.24	.81	−.02
Individual difference measures						
Decision regret	.22***	.05	.10*	2.07	.48	.15***
Positive outcome focus	.10*	.10	.04	2.08	.48	.06
Negative outcome focus	.14***	.11	.02	3.16	.32	N/A
General outcome focus	.02	.21	.02	1.81	.55	.00
Consideration of future outcomes	−.18***	.03	−.04	1.70	.59	−.05
Susceptibility to scams	.34***	.20	.16**	2.82	.35	N/A
Financial knowledge	−.21***	.05	−.03	1.82	.55	−.07
Financial risk tolerance	.20***	.11	.05	1.85	.54	.05
History of financial fraud: yes	.04	.00	−.06	1.89	.53	−.04
Has received scam IRL: yes^§^	−.22***	.05	N/A	N/A	N/A	N/A
Has responded to scam IRL: yes^¶^	.39***	.99	.09	1.75	.57	.12**
Fuzzy‐trace theory measures						
Categorical risk	−.12**	.22	−.05	1.47	.68	−.07
Global risk	−.16***	.05	−.05	1.25	.80	−.07*
Gist principles	.02	.00	.03	1.06	.94	.02
Specific risk	.28***	.09	−.02	2.70	.37	N/A
Quantitative risk	.17***	.11	.05	2.54	.39	N/A
Scam‐related measures						
Perceived risks	−.41***	.17	−.15***	1.32	.76	−.15***
Perceived benefits	.74***	.55	.52***	1.59	.63	.53***
Letter is genuine: yes	.48***	.23	.05	1.78	.56	.09
Constant	N/A	N/A	.00	N/A	N/A	.00
*Pseudo‐R^2^*			1.00			1.00

Abbreviations: N/A, not applicable; IRL, in real‐life; Tol., tolerance; VIF, variance inflation factor.

^†^Education was coded as 1 = do not have high school degree or GED, 2 = high school degree/GED, 3 = associate's degree, 4 = bachelor's degree, 5 = master's degree, 6 = professional degree (MD, JD, etc.), 7 = PhD.

^‡^Income was coded as 1 = $0–$24,999; 2 = $25,000–$49,999; 3 = $50,000–$74,999; 4 = $75,000–$124,999; 5 = $125,000–$174,999; 6 = $175,000+.

^§^Variable omitted for joint entry regression model.

^¶^ *N* for this variable is 369 (No: *n* = 319, Yes: *n* = 50).

^*^ *p* < .05.; ^**^ *p* < .01.; ^***^ *p* < .001.

Demographic variables: Non‐White and more conservative participants were more inclined to respond to the solicitation. To better understand the role of race/ethnicity, we separately compared White participants (73%) to other racial/ethnic groups. Only identifying as Black or African American (12%) predicted willingness to contact the scammers (*β* = .18, *p* < .001, *Pseudo‐R^2^* = .53).

Individual difference measures: Stronger contact intentions were observed among participants who scored higher on assessments of decision regret, positive outcome focus, negative outcome focus, susceptibility to scams, and financial risk tolerance or had previously responded to a real‐life scam. Weaker intentions were observed among participants who scored higher on assessments of future outcome considerations and financial knowledge, as well as those participants who had previously received a real‐life scam offer.

Fuzzy‐trace theory measures: As predicted, participants reported lower contact intentions when they scored higher on measures of gist‐based risk perception (categorical and global risks). Conversely, participants reported higher contact intentions when they scored higher on measures of verbatim‐based risk perception (specific and quantitative risks). Endorsement of scam‐related gist principles did not predict willingness to respond to the solicitation.

Scam‐related measures: Participants reported a higher likelihood of complying with the scammers when they perceived the offer as less risky, more beneficial, and as genuine.

#### 3.2.2 Joint entry

We next conducted a multiple regression analysis in which all predictors were entered jointly (*Pseudo‐R^2^* = 1.00, Table 3, middle columns). (Note that the question of whether participants had received a real‐life scam offer was omitted in favor of the question whether participants had responded to a real‐life scam offer, as the latter was dependent on participants’ response to the former). In this joint model, decision regret, prior susceptibility to scams, lower risk perceptions, and higher benefit perceptions predicted a higher willingness to contact the scammer.

Because negative outcome focus, self‐rated susceptibility to scams, specific risk, and quantitative risk all yielded variance inflation factors >2.50, we re‐ran the full model displayed in Table 3 (middle columns) and excluded these variables (Table 3, right columns, *Pseudo‐R^2^* = 1.00). In this reduced model, having previously responded to real‐life scams now significantly predicted intentions to contact the scammers in our study, as did global risk perception.

## 4 DISCUSSION

The present study evaluated which theoretically and empirically derived explanatory factors render individuals vulnerable to MMS, and whether susceptibility to scams can be altered by influencing verbatim‐ and gist‐based processing of scam‐related risks.

### 4.1 The role of risks, benefits, and perceived genuineness

Although 75% of participants identified the solicitation as fraudulent, 43% of participants considered contacting the scammers. While high, our results match those of earlier investigations (Modic & Lea, 2013; Titus et al., 1995; Wood et al., 2018). In line with previous work (Hanoch et al., 2006; Wood et al., 2018), we also demonstrate that stronger contact intentions are tied to perceptions of high benefits and low risks. Highlighting the psychological underpinnings of susceptibility, the scam message did not vary but its perceived benefits and risks varied considerably across individuals. MMS are likely designed to evoke perceptions that benefits are well‐defined and seemingly high (i.e., earning $500,000), whereas their risks—or costs—are ill‐defined or appear small (Button, McNaughton Nicholls, et al., 2014) but even our more “neutral” scam message evoked such perceptions in the vulnerable. However, overall, contact intentions drop when individuals are asked to pay a fee to “activate” their prize, with higher activation fees (i.e., $100) resulting in higher risk perceptions than lower activation fees (i.e., $5; Wood et al., 2018).

Our solicitation was also more successful when it was perceived as an authentic rather than dubious offer. This finding aligns with past research suggesting that both perceived genuineness and authenticity affect scam susceptibility (Button, McNaughton Nicholls, et al., 2014). Believing that such offers are authentic can be thought of as an initial sign of falling for a scam and should also reflect factors related to scam susceptibility, as shown in Table S1.

### 4.2 The role of individual difference measures

A few individual‐difference measures predicted compliance intentions once all other factors were taken into consideration. These stable predictors included the tendency to anticipate regret for forgone opportunities, that is, wondering what would have happened if one had chosen a different option (Nordgren et al., 2007; Richard et al., 1996). Because many scam letters tend to induce a sense of urgency and anticipated regret in order to increase compliance (Chang, 2008), this novel finding about decision regret warrants more attention in future MMS studies.

Surveys suggest that individuals who have been previously victimized are more—rather than less—likely to fall for other scams as well (Titus et al., 1995). This corresponds to our finding that those participants who self‐report higher levels of fraud susceptibility or have experience responding to solicitations in real life were especially likely to respond to the solicitation in our experiments as well (Schoepfer & Piquero, 2009; Titus & Gover, 2001), even when accounting for other factors. Similarly, it echoes research showing that past online risk‐taking is associated with stronger intentions to take online risks in the future (White et al., 2015). Presumably, those who took these risks were likely to have experienced negative outcomes, and their apparent failure to learn from experience raises important questions about purely reinforcement‐learning accounts of risky decision making. In this context, perceptions, rather than experienced outcomes, seem to better explain risk‐taking.

### 4.3 The role of verbatim‐ and gist‐based processing

The present study is among the first to attempt to systematically alter susceptibility to MMS. Consistent with fuzzy‐trace theory, we found that gist‐based risk processing was associated with decreased willingness to contact the scammers, whereas the opposite was true for verbatim‐based processing (White et al., 2015). As such, fuzzy‐trace theory presents an attractive starting point for the development of interventions aimed at decreasing susceptibility to MMS.

Contrary to expectations, the manipulation of risk processing did not change decision makers’ perception of the solicitation offer. Consistent with the expected direction of results, participants randomized to the gist condition were somewhat more likely to identify the solicitation as risky, and as a scam, than participants in the verbatim condition. However, these differences were not statistically significant with nonparametric tests, and contact intentions did not vary between groups. It is possible that our gist and verbatim conditions were ultimately too subtle to influence participants’ responses to the scam letter: Both of the programs on which our gist condition was based (Anand et al., 2011; Cook et al., 2014) required several hours of instruction. Although gist‐based reasoning can benefit from as little as a 1‐h training session (Wolfe et al., 2015), our very brief, one‐time intervention might have been too superficial to achieve the desired effectiveness. Alternatively, it is possible that the gist and verbatim conditions led to similar outcomes because participants in both conditions encoded the same message: Participants, on average, in both conditions might have come to the conclusion that risks were similarly of “medium” magnitude (Table 1).

### 4.4 Constraints on generality

The present study is not without other limitations. First, it should be noted that our experiment did not feature a control group. Consequently, we were unable to evaluate the relative effectiveness of the verbatim and gist conditions on participants’ risk‐benefit perceptions and willingness to comply with the solicitation. We also cannot determine whether both groups might have differed from a non‐intervention control simply because they paid more attention to the message.

Second, the present study only asked participants to indicate their intentions to comply with the scammers and could not verify whether participants would have indeed contacted the MMS telephone number to claim their prize. That being said, our results mirror earlier work using similar sources of participants as well as research conducted with diverse populations (e.g., Modic & Lea, 2013). In addition, our investigation employed an MMS that is typically delivered via snail mail. This could have impacted the credibility of our solicitation, although most scams nowadays are delivered via electronic means rather than snail mail (Anderson, 2013). Future studies should attempt to elicit actual responses while maintaining ethical standards.

### 4.5 Conclusions

Although the present study confirmed that fuzzy‐trace theory measures correlated with decision makers’ willingness to engage with scams, a manipulation grounded in this framework did not change intentions to respond to a letter scam. Considering that past experiences with fraud victimization did not deter participants’ willingness to contact the scammers in the present study either, it appears that susceptibility to scams could be difficult to alter.

## CONFLICT OF INTEREST

The authors declare no conflict of interest.

### PEER REVIEW

The peer review history for this article is available at https://publons.com/publon/10.1002/brb3.2391

## Supporting information

Supporting Information

## Data Availability

Upon reasonable request addressed to Julia Nolte (jn472@cornell.edu), data can be made available to other authors.
