# Supplementary material for: Compliance with mass marketing solicitation: The role of verbatim and gist processing
Source: Brain Behav. 2021 Oct 17;11(11):e2391. doi: 10.1002/brb3.2391 (PMC8613425; doi:10.1002/brb3.2391)
Supplement: Supplementary file 1 — Supporting Information [file BRB3-11-e2391-s001.docx]

**Appendix**

**Appendix A: Study Materials**

**Appendix B: Supplementary Analyses**

**Appendix A: Study Materials**

**Verbatim condition**

In the next page, you will be presented with a letter. While reading the letter, focus on the

exact letter details. Consider the following:

- Who organized the contest?
- How big is the prize money you have won?
- What do you need to do to claim your winnings?
- When is the deadline?

While reading the paper, you should focus on. Please tick all that apply:

___ Who organized the contest?

___ Who organized the contest?

___ What do you need to do to claim your winnings?

___When is the deadline?

**Gist condition**

In the next page, you will be presented with a letter. While reading the letter, imagine you

want to tell a friend or family member about it. Consider the following:

- What is the gist of the letter?
- How would you summarize the content of the letter in one or two sentences, using your own words?

According to the above, what should you consider when reading the letter? Please tick all

that apply:

___ The gist of the letter

___ A summary of the letter

___ You should use your own words

**Categorical Risk Taking**

| Strongly disagree |  |  |  | Strongly agree |
| --- | --- | --- | --- | --- |
| 0 | 1 | 2 | 3 | 4 |

- If you give out your personal details over the phone, risks will add up and you WILL experience identify theft and financial loss.
- When in doubt about giving your personal information over the phone, it is better to play it safe.
- If you give out your personal details over the phone to people you do not know, risks will add up and you WILL experience identity theft and financial loss.
- Even if giving personal information over the phone seems like a low risk activity, it will add up to 100% if you do it often.
- Giving your personal information over the phone even ONCE can lead to identity theft and financial loss.
- Even low risks, such as identity theft, happen to people.
- Claiming prizes over the phone can be risky, and could result in identity theft and financial loss.
- Once someone has your personal details, there is no second chance.
- If you cannot protect your personal information, you should avoid giving it out over the phone.

**Global Risk Taking**

| None | Low | Medium | High |
| --- | --- | --- | --- |
| 0 | 1 | 2 | 3 |

- Overall, for you, which best explains the risks of giving out your personal information over the phone?
- Overall, for you, which best explains the risks of claiming prizes you receive over the phone?

**Gist Principles**

- Better not to claim prizes over the phone, than risk having your identity stolen and experiencing financial loss.
- Better to be safe than sorry.
- Better to never give out personal information over the phone than risk having your identity stolen.
- Better to wait giving out personal information over the phone, when you are not 100% sure who you are speaking to.
- I have a responsibility to my family to not give out my personal details to people over the phone.
- Better not to claim prizes I have never heard about before than to hurt my family.
- I have a responsibility to myself to keep my personal details safe.
- Avoid risk.
- Better to claim prizes over the phone as the risks are low. (R)
- Prizes collected over the phone are always safe to claim. (R)
- Claiming prizes over the phone is better than not winning prizes at all. (R)
- Claiming prizes is worth the risks of giving personal information over the phone. (R)
- Giving out my personal information to claim prizes is worth the risk of identity theft. (R)

**Specific Risk**

| Very unlikely |  |  |  |  |  |  |  | Very likely |
| --- | --- | --- | --- | --- | --- | --- | --- | --- |
| 0 |  | 1 |  | 2 |  | 3 |  | 4 |

- I am likely to have my personal details stolen and used against me within the next 6 months
- I am likely to be taken advantage of over the phone in the next 6 months by a person I do not know

**Quantitative Risk**

- To the best of your knowledge, have you had your personal information stolen? (0% - 100%)
- To the best of your knowledge, have you been a victim of a financial scam or a fraud? (0% - 100%)

**Appendix B: Supplementary Analyses**

**Table S1**

*Regression Results Predicting Genuineness Perceptions for Each Variable Entered Separately versus All Variables Entered Jointly*

|  | ***Separate Entry*** | | ***Joint Entry*** | | | ***Joint Entry (Removing High VIF)*** |
| --- | --- | --- | --- | --- | --- | --- |
| **Variables** | ***b*** | ***Pseudo-R^2^*** | ***b*** | ***VIF*** | ***Tol.*** | ***b*** |
| **Demographic variables** |  |  |  |  |  |  |
| Age | -.02** | .03 | .03 | 1.97 | .51 | .03 |
| Gender: female | -.41* | .02 | -.24 | 1.24 | .81 | -.37 |
| Race/ ethnicity: non-White | .60** | .02 | .21 | 1.70 | .59 | .43 |
| Employment status: not full-time | -.85*** | .04 | -.75 | 1.28 | .78 | -.83 |
| Education^†^ | .19* | .01 | -.10 | 1.68 | .59 | -.08 |
| Income^‡^ | .11 | .00 | .14 | 1.45 | .69 | .14 |
| Marital status: not married | -.43* | .01 | -.03 | 1.38 | .72 | -.17 |
| Political worldview | .22*** | .06 | .16 | 1.15 | .87 | .15 |
| **Individual difference measures** |  |  |  |  |  |  |
| Decision regret | .21** | .02 | -.32 | 2.69 | .37 | N/A |
| Positive outcome focus | .03** | .01 | .05 | 1.96 | .51 | .16 |
| Negative outcome focus | .24*** | .08 | .34 | 3.51 | .29 | .34 |
| General outcome focus | .06 | .11 | .04 | 2.21 | .45 | -.14 |
| Consideration of future outcomes | -.75*** | .07 | .14 | 2.17 | .46 | .04 |
| Susceptibility to scams | .85*** | .27 | .65* | 2.68 | .37 | N/A |
| Financial knowledge | -.50*** | .10 | -.06 | 2.21 | .45 | -.18 |
| Financial risk tolerance | .45*** | .14 | .50* | 1.67 | .60 | .59** |
| History of financial fraud: yes | .28 | .00 | -.44 | 1.99 | .50 | -.04 |
| Has received scam IRL: yes^§^ | -.82*** | .04 | N/A | N/A | N/A | N/A |
| Has responded to scam IRL: yes^¶^ | 2.79*** | .85 | .48 | 1.72 | .58 | .81 |
| **Fuzzy-trace theory measures** |  |  |  |  |  |  |
| Categorical risk | -.54** | .13 | -.45 | 1.70 | .59 | -.78 |
| Global risk | -.57*** | .07 | -.40 | 1.30 | .77 | -.45 |
| Gist principles | -.07 | .00 | .04 | 1.16 | .87 | .01 |
| Specific risk | .75*** | .18 | -.13 | 3.46 | .29 | N/A |
| Quantitative risk | .02*** | .10 | .01 | 2.96 | .34 | N/A |
| **Scam-related measures** |  |  |  |  |  |  |
| Perceived risks | -.35*** | .10 | -.27 | 1.51 | .66 | -.25 |
| Perceived benefits | .53*** | .25 | .26 | 2.20 | .46 | .23 |
| Intentions to call number | .09*** | .33 | .10 | 2.45 | .41 | .15 |
| **Constant** | N/A | N/A | -6.25 | N/A | N/A | -3.95 |
| ***Pseudo-R^2^*** |  |  | .94 | | | .93 |

*Note*. Logistic regression with unstandardized regression weights, predicting perceptions that the letter is genuine (0 = letter is not genuine, 1 = letter is genuine).^†^ Education was coded as 1 = Do not have high school degree or GED, 2 = High school degree/GED, 3 = Associate’s degree, 4 = Bachelor’s degree, 5 = Master’s degree, 6 = Professional degree (MD, JD, etc.), 7 = PhD. ^‡^ Income was coded as 1 = $0 – $24,999, 2 = $25,000 – $49,999, 3 = $50,000 – $74,999, 4 = $75,000 – $124,999, 5 = $125,000 – $174,999, 6 = $175,000+. ^§^ Variable omitted for joint entry regression model. ^¶^ *N* for this variable is 369 (No: *n* = 319, Yes: *n* = 50)*.*  IRL = in real-life. VIF = Variance inflation factor. Tol. = Tolerance. N/A = not applicable. * *p* < .05, ** *p* < .01, *** *p* < .001.
